# Prediction of coronary artery lesions in children with Kawasaki syndrome based on machine learning

**DOI:** 10.1186/s12887-024-04608-2

**Published:** 2024-03-05

**Authors:** Yaqi Tang, Yuhai Liu, Zhanhui Du, Zheqi Wang, Silin Pan

**Affiliations:** 1 Heart Center, Qingdao Women and Children’s Hospital, Qingdao University, Qingdao, China; 2 Dawning International Information Industry Co., Ltd., No. 78 Zhuzhou Road, Laoshan District, Qingdao, China; 3 Sugon Nanjing Institute, Co., Ltd., No. 519 Chengxin Avenue, Fangyuan Road, Jiangning District, Nanjing, China; 4 School of Mathematics, Jilin University, Changchun, China

**Keywords:** Kawasaki syndrome, Coronary artery lesions, Machine learning, Random forest

## Abstract

**Objective:**

Kawasaki syndrome (KS) is an acute vasculitis that affects children < 5 years of age and leads to coronary artery lesions (CAL) in about 20-25% of untreated cases. Machine learning (ML) is a branch of artificial intelligence (AI) that integrates complex data sets on a large scale and uses huge data to predict future events. The purpose of the present study was to use ML to present the model for early risk assessment of CAL in children with KS by different algorithms.

**Methods:**

A total of 158 children were enrolled from Women and Children’s Hospital, Qingdao University, and divided into 70–30% as the training sets and the test sets for modeling and validation studies. There are several classifiers are constructed for models including the random forest (RF), the logistic regression (LR), and the eXtreme Gradient Boosting (XGBoost). Data preprocessing is analyzed before applying the classifiers to modeling. To avoid the problem of overfitting, the 5-fold cross validation method was used throughout all the data.

**Results:**

The area under the curve (AUC) of the RF model was 0.925 according to the validation of the test set. The average accuracy was 0.930 (95% CI, 0.905 to 0.956). The AUC of the LG model was 0.888 and the average accuracy was 0.893 (95% CI, 0,837 to 0.950). The AUC of the XGBoost model was 0.879 and the average accuracy was 0.935 (95% CI, 0.891 to 0.980).

**Conclusion:**

The RF algorithm was used in the present study to construct a prediction model for CAL effectively, with an accuracy of 0.930 and AUC of 0.925. The novel model established by ML may help guide clinicians in the initial decision to make a more aggressive initial anti-inflammatory therapy. Due to the limitations of external validation and regional population characteristics, additional research is required to initiate a further application in the clinic.

**Supplementary Information:**

The online version contains supplementary material available at 10.1186/s12887-024-04608-2.

## Introduction

Kawasaki syndrome (KS) is a mucocutaneous lymph node syndrome associated with vascular endothelial dysfunction and immune activation that mainly affects children under the age of 5 [1]. The disease is described in all continents but presents the highest annual incidence in Asian countries [2–3]. The incidence of KS in children under 5 years was 68.8 to 107.3 per 100,000 children from 2013 to 2017 in Shanghai [4]. The incidence of KS in children aged 0–4 years was 309 to 330.2 per 100,000 children from 2015 to 2016 in Japan [5]. Coronary artery lesion (CAL) is the most serious complication in the acute phase of KS, including coronary artery dilatation (CAD), coronary artery aneurysm (CAA), long-term coronary artery stenosis (CAS), and even myocardial infarction (MI) [6]. The latest national survey conducted by the Japan Circulation Society (JCS) shows that 7% of children with KS present vascular complications in the acute phase, and 2.3% develop cardiovascular sequelae after discharge [7]. About 20-25% of untreated cases will develop into severe CAL, which makes KS the most common cause of pediatric acquired heart disease in developed countries [8]. Therefore, it is necessary to carry out CAL risk prediction stratification for early diagnosis and intensive care.

Machine learning (ML) is a subset of artificial intelligence (AI), which identifies the features of data sets by constructing corresponding algorithms [9]. The programmer works to find out the characteristics of the features related to the outcome of each event and establishes the training set. The machine determines what characteristics each outcome is related to by learning the training set. In brief, it identifies which end category it belongs to when there is an unfamiliar string of data. With the size of the training sets increasing, the accuracy of ML improved gradually. ML has been extensively used in the field of medicine and health care. Takeuchi al [10]. used a random forest (RF) classifier to establish a prediction model for intravenous immunoglobulin (IVIG) resistant KS with a sensitivity of 79.7% and a specificity of 89.3%, which was significantly better than other traditional models. Xue et al. [11] established a Cox model to predict the risk associated with blood lipid profiles (Lp) in children with ST-segment elevation myocardial infarction (STEMI). It is pointed out that children with STEMI presented higher Lp (a) and lower HDL-C, as well as apoA1, which are more likely to have a higher risk of adverse cardiovascular disease. Sun et al. [12] designed a prediction model based on RF to estimate the probability of arrhythmia after transcatheter closure of atrial septal defect (ASD). Li et al. [13] used manual feature engineering to identify the degree of bone marrow invasion of acute myeloid leukemia (AML), and the sensitivity and specificity of the model were 87.6% and 89.5%, respectively.

The purpose of the present study was to predict who among patients with KS will develop into severe CAL by constructing corresponding algorithms in ML based on clinical manifestation and clinical auxiliary examination.

## Methods

### Study population

Abstracted data from the eligible patients in Women and Children’s Hospital, Qingdao University from May 2021 to June 2022 including patients who received a diagnosis of KS or patients with incomplete KS (IKS) or IVIG-resistant KS under 5 years old at enrollment. The 6th edition diagnostic guidelines revision was used as a guide in the present study [14]. To eliminate irrelevant clinical manifestations and make sure all patients were treated with the same therapy, excluded patients with infections or immunodeficient disease, or who did not receive IVIG within 10 days from the onset. Patients treated with glucocorticoids were also eliminated since the influence of glucocorticoids is not clear yet (15–16). Besides, the medical records with a missing value ratio > 70% were excluded. Echocardiography (Echo) was performed to evaluate the coronary artery before admission and discharge. Compared with the defined internal diameter measurement previously [17], coronary artery abnormality was defined as a Z-score of the coronary artery internal diameter of 2.5 or more according to the 6th edition diagnostic guidelines [14]. Patients at enrollment all accepted a continuous intravenous infusion of IVIG 2 g/kg/24 hours combined with oral aspirin 30 mg/kg/d from the onset, and 3 mg/kg/d after the normalization of C-reactive protein (CRP) and body temperature.

### Feature vectors selected

Feature selections combined both clinical manifestations of KS and the high-risk factors of CAL confirmed by previous studies [18–21], excluding the unconventional clinical auxiliary examination. All features were collected before patients were given IVIG treatment. Medical records with missing counting features such as height and weight were analyzed by linear regression analysis and filled according to the test regression equation. The qualitative features of clinical manifestations were transformed into counting features. The K-nearest neighbor (KNN) filling method was used to complete the missing qualitative features or correct the outliers through the correlation of the data in each dimension.

### Dimensionality reduction

The numerical features were standardized in one dimension and carried on the principal component analysis (PCA) of the standardized data, which aims to reduce dimensionality and extract features [22]. The high correlation between features had been eliminated. PCA simplified the complexity of analysis but kept the original information, and integrated multiple vectors into a minority of comprehensive vectors. The total variance of P random features was divided into the sum of the variance of P unrelated random features and kept the first principal component maximum. The variance contribution rate was defined as the ratio of the variance of the principal component to the total variance, and the higher the value, the more original information was reflected in the component. The weight of the vectors was equal to the variance contribution rate of the principal component, and to make the sum of weights of coefficient of the vectors in the linear combination of principal components was 1 by keeping uniformization of the weighted average.

### Artificial data synthesis

The SMOTE was proposed by Chawla [23] in 2002, which synthesized better classifier performance by using the interpolation method combining the over-sampling of the minority class and the under-sampling of the majority class. N was the number of the minority classified in the training set, the process was as follows:

1. Sampled ${x_i}$ from the minority class. Its eigenvector was ${x_i},\,i\, \in \{ 1,...,N\}$. First of all, the K nearest neighbors of the sample ${x_i}$ were found from all the N samples of the minority class. Marked it as ${x_{i(near)}},near \in \{ 1,...,K\}$.
2. Then sampled randomly from the K neighbors and regenerated into a random number $\mu$ from 0 to 1, thus a new sample ${x_{i(near)}}$ was synthesized: ${x_{new}}\, = \,\,{x_i}\, + \,\mu ({x_{i(k)}} - {x_i}$
3. Synthesized T new samples by repeating steps 1 and 2 for T times.

### Model building and verification

The software VScode was employed for programming and Python version 3.6 (Python Software Foundation) was performed for statistical analyses. Packages as sklearn 1.2.2, pandas 2.1.3, and numpy 1.24.3 were used to add 95% confidence intervals (CI) and complete cross-validation.

To avoid overfitting, the validation set approach was performed. The entire data sets were divided into 70% and 30% as the training sets and the test sets. The classifiers RF, logistic regression (LR), and eXtreme Gradient Boosting (XGBoost) were constructed using demographic variables processed by the input features of the training sets severally. The data from test sets were substituted into the models for validation. According to the prediction results of the models, the data was calculated one by one as positive examples in this order and figured out the values of two important quantities each time. The receiver operating characteristic (ROC) curve was obtained by arranging the two quantities as horizontal and vertical coordinates respectively. The discriminatory capacity of the model was assessed using the area under the ROC curve (AUC). The vertical axis of the ROC curve was sensitivity or true positive rate (TPR), while the horizontal axis was false positive rate (FPR). Besides, the five-fold cross validation method was used through all the data to avoid overfitting. The entire data set was divided into five parts and each set divided contains the full category of labels. The cross_val_score function is used to assess the accuracy of the model. The 2x standard deviation method was used to calculate the boundary of the confidence interval. The average value of accuracy minus 2x standard deviation to calculate lower bounds and with the average value plus 2x the standard deviation to calculate the upper bound. Results are expressed as an odds ratio with a 95% confidence interval (CI).

## Results

### Demographic characteristics

A total of 158 children were enrolled in the present study, including 104 males (65.8%). The mean age was 779.91 ± 654.63 days. 9.49% (*n* = 15) of children were diagnosed as CAL according to the Z-score assessment by the Echo. 1.26% (*n* = 2) of the research population present IKS and 1.26% (*n* = 2) occurred IVIG-resistant KS. The detailed baseline characteristics are shown in Table 1.

**Table 1:** Demographics of research population. *N* = 158

Characteristic		Characteristic	n (%)
Height (cm)	87.32 ± 16.63	Male	104 (65.82)
Weight (kg)	12.88 ± 7.10	Rash	
Age (d)	779.91 ± 654.6	macular papule	104 (65.82)
Hospitalization time (d)	7.35 ± 4.57	millet	18 (11.39)
Fever Time (d)	6.09 ± 2.97	Bulbar conjunctival injection	144 (91.14)
WBC (×10^9^)	14.95 ± 5.56	Chapped lips	68 (43.04)
Hb (G/L)	111.52 ± 13.93	Reddening of lips	142 (89.87)
CRP (mg/l)	67.60 ± 50.13	Strawberry tongue	125 (79.11)
PLT(×10^9^)	331.74 ± 143.82	Cervical lymphadenopathy	121 (76.58)
ESR (mm/h)	62.04 ± 25.21	Changes in peripheral extremities	135 (85.44)
Na^+^ (mmol/l)	136.57 ± 3.25	Changes of Perineal	36 (22.78)
ALT (U/L)	56.60 ± 91.64	Z-sroce > 2.5	15 (9.49)
AST (U/L)	47.98 ± 83.73	Arrhythmia	73 (46.20)
LDH (U/L)	294.85 ± 72.02	IKS	2 (1.26)
CK (U/L)	54.96 ± 38.85	IVIG-resistant KS	2 (1.26)
CK-MB (U/L)	17.99 ± 21.24		
cTNI (ng/ml)	0.15 ± 0.89		
PCT (n/ml)	1.14 ± 1.66		

WBC: White blood cell count; Hb: Hemoglobin; PLT: Platelet count; CRP: C-reactive protein; ESR: Erythrocyte Sedimentation Rate; Na^+^: Serum sodium concentration; ALT: Alanine aminotransaminase; AST: aspartate aminotransferase; LDH: lactate dehydrogenase; CK: creatine kinase; CK-MB creatine kinase-MB; cTNI: cardiac troponin I; PCT: procalcitonin

### Predictive model for CAL

**Fig. 1:**
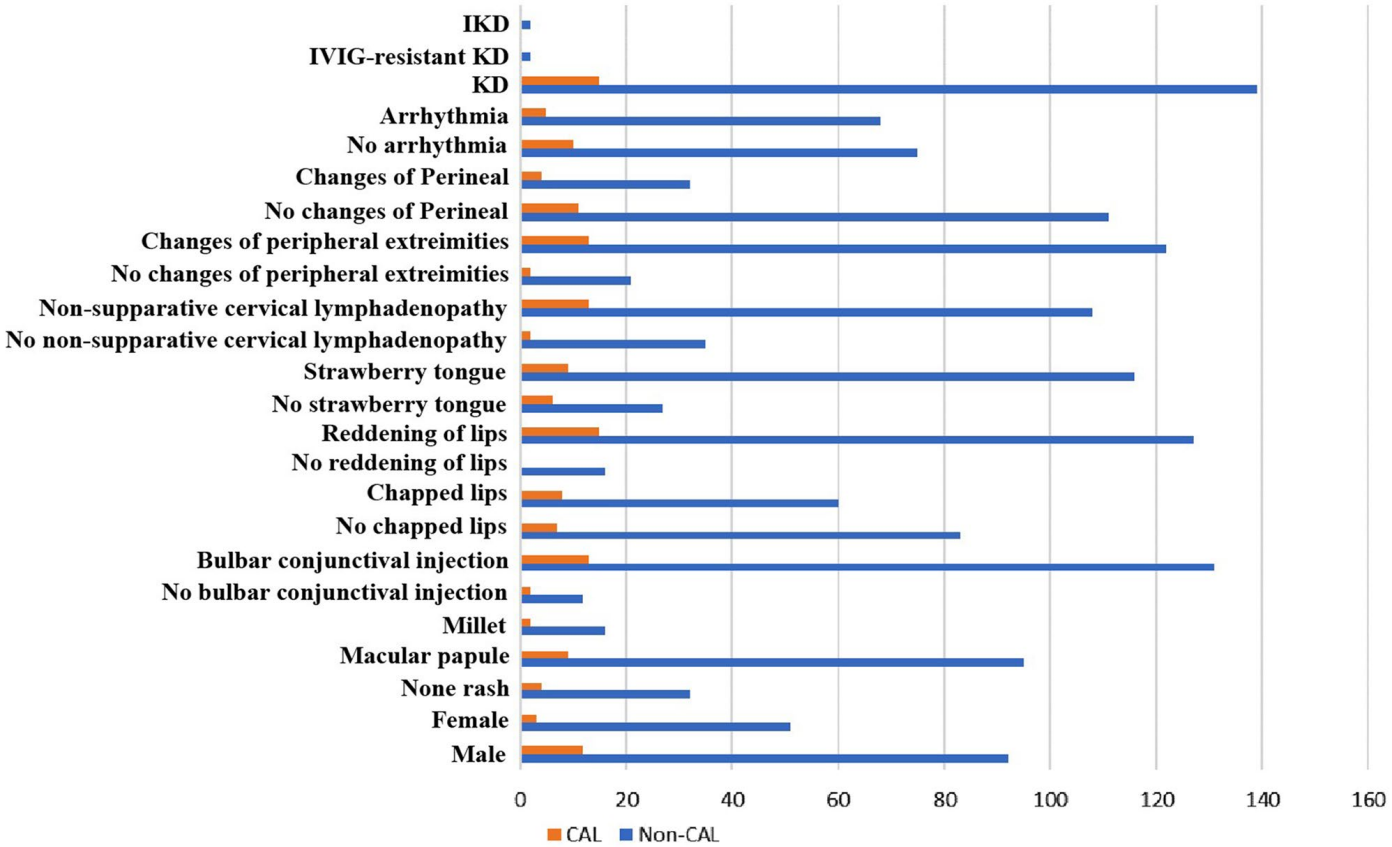
Comparison between Non-CAL patients and CAL patients in the input qualitative variables

**Fig. 2:**
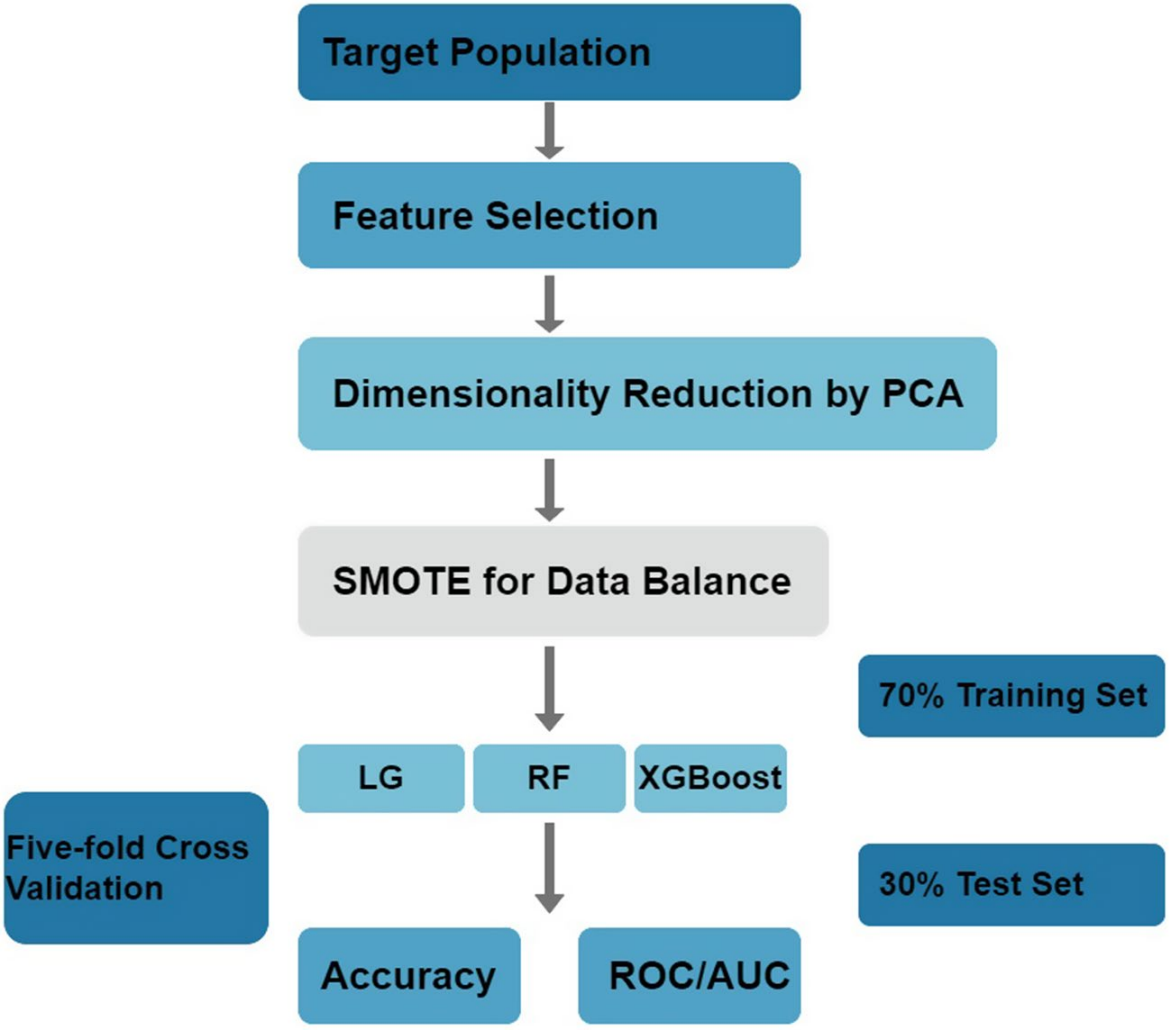
Schematic diagram for model building

**Fig. 3:**
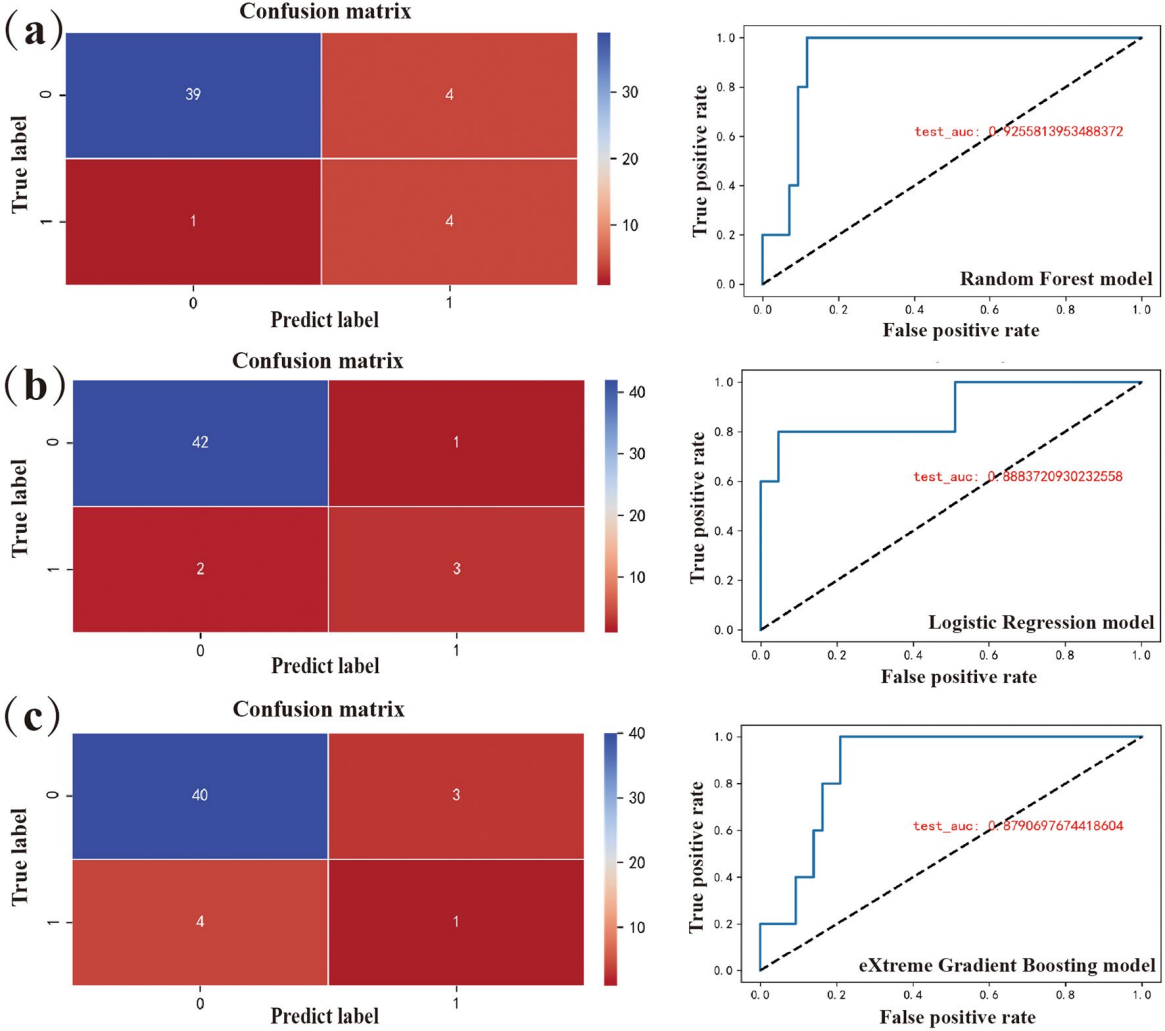
Demonstration of the prediction effect of the 3 models. (**a**) Demonstration of the prediction effect of RF model. (**b**) Demonstration of the prediction effect of LG model. (**c**) Demonstration of the prediction effect of XGBoost model

The cohort for the model included demographic characteristics, signs, symptoms of KS, laboratory results, and diagnosis. The comparison between Non-CAL and CAL in the input qualitative variables is shown in Fig. 1. After excluding the variables that were not common in the clinical routine examination and deleting data that did not meet the requirements, 29 feature vectors were determined (Table S1). After PCA dimension reduction, 24 principal components are retained (not shown in the Figures). For solving the imbalance between the data, the SMOTE was used for equalization and the distribution of the training set after the data balance was shown in Table 2. The SMOTE was used for the construction of classifiers from imbalanced data consisting of 143 labels as non-CAL and 15 labels as CAL. There were 110 records in the training set, including 100 children with non-CAL and 10 with CAL. There were 48 records in the test set, including 43 children with non-CAL and 5 with CAL. The schematic diagram of the prediction model conduction is shown in Fig. 2. The AUC of the RF model was 0.925 according to the validation of the tests set and the confusion matrix was shown in Fig. 3. The accuracy obtained from 5-fold cross validation was 0.947, 0.868, 0.972, 0.945, 0.919 and 0.930. The average accuracy was 0.930 (95% CI, 0.905 to 0.956). The AUC of the LG model was 0.888 and the average accuracy was 0.893 (95% CI, 0,837 to 0.950). The AUC of the XGBoost model was 0.879 and the average accuracy was 0.935 (95% CI, 0.891 to 0.980). To show the several results distinctly, we established data collection in Table 3.

**Table 2:** Distribution before and after SMOTE

Category	Non-CAL	CAL
Proportion before SMOTE	0.91	0.09
Proportion after SMOTE	0.5	0.5

**Table 3:** Demonstration of the prediction effect of the 3 models

Model	AUC	Average Accuracy	95% CI	Accuracy
RF	0.925	0.930	0.905–0.956	0.947/0.868/0.972/0.945/0.919
LG	0.888	0.893	0.837–0.950	0.921/0.842/0.891/0.919/0.919
XGBoost	0.879	0.935	0.891–0.980	0.921/0.973/0.946/0.918/0.919

## Discussion

Prevention of CAL is a significant step in the treatment of KS. The standard therapy for KS is effective in reducing the incidence of CAL significantly [24]. However, even with timely initiation of IVIG treatment, coronary artery dilatation might occur in 30% of children, and 5–10% of children might eventually develop permanent coronary artery disease [25]. Early identification and possible intervention might reduce the risk of late coronary artery dilatation. It has been confirmed that corticosteroid therapy combined with IVIG as the initial treatment may intervene in CAL effectively in children with severe KS or IVIG-resistant [26–28]. However, it remains controversial whether corticosteroids should be used as initial therapy [25]. One of the implications of our predictive algorithm is to recognize patients with a high risk of CAL in the early stage of the disease, which can help guide clinicians in the initial decision to make a more aggressive initial anti-inflammatory therapy. For patients with a high risk for CAL sought to consider a careful follow-up such as more frequent echocardiography.

There were several predictive models with discriminative results established by researchers in different countries or regions for CAL children and IVIG-resistant KS. Chang et al. [29] established a scoring system based on the CRP, neutrophil/lymphocyte ratio, male gender, and IVIG resistance with a sensitivity and a specificity of 60.8% and 70.6%. Hua et al. [30] developed a CAL risk prediction model in children under 6 months of age. The AUC of the model was 0.731, with a sensitivity and specificity of 64.7% and 80.9%. Lee et al. [31] used N-terminal-pro-brain natriuretic protein (NT-proBNP) and polymorphonuclear Neutrophil (PMN) to create the prediction model for CAL, which presents a sensitivity of 73.3% and a specificity of 67.9%. In China, Yang et al. [32] constructed a predictive tool for the efficacy of IVIG therapy in children with KS, and the sensitivity and specificity were 56% and 79% in the internal verification, respectively. However, these published risk assessment systems scoring of IVIG-resistance or CAL were short of the external data verification (33–34).

The traditional models are constructed with only a few features, while the model calculated by ML can integrate all aspects of clinic feature vectors. ML is suitable for many tasks and makes it easy for the model to retrain and update using the newest data [35]. Supervised learning in ML identifies the relationship between input data and output data in the training set, then summarizes the new data into a known label according to its features, which one we choose to employ (36–37). The input data used in the present study covered common clinical manifestations and auxiliary examinations based on the latest 6th edition diagnostic guidelines, which makes the clinical application of the model easier. In the present study, three classifiers were employed to establish the model. According to the result of validation studies, the accuracy of the RF model was more than 0.90 and the AUC was 0.925. The accuracy of the XGBoost model was 0.930 with a 0.879 AUC. The result of validation showed that our model has feasibility and application prospects in the clinical risk prediction of CAL. In addition, the validation set approach and five-fold cross validation method were performed throughout the process of model establishment and validation. These efforts allow us to avoid the problem of overfitting to the greatest extent and increase the credibility of the model.

This study has several limitations. A more stringent external validation from other institutions should be involved in further assessing the generalizability of the proposed scoring model before applying it. Besides, we have not subsumed hypoalbuminemia and hyperbilirubinemia which are newly added in the sixth edition of the guidelines in the features. The population studied in the present study appears strongly regional characteristics, which might represent the patient population in the east of China.

## Conclusion

Three different algorithms were used in the present study to construct a prediction model for CAL effectively. The accuracy of the RF model was more than 0.90 and the AUC was 0.925, which shows that our model has feasibility and application prospects in the clinical risk prediction of CAL. The novel model established by ML may help guide clinicians in the initial decision to make a more aggressive initial anti-inflammatory therapy. Compared with models established by traditional methods in other regions of China, the verification results of this model showed higher accuracy. Due to the limitations of external validation and regional population characteristics, additional research is required to initiate a further application in the clinic.

### Electronic supplementary material

Below is the link to the electronic supplementary material.

Supplementary Material 1: Numerical range of input features

## Data Availability

All data generated or analyzed during this study are included in this published article.

## Abbreviations

AI: artificial intelligence
ASD: atrial septal defect
AML: acute myeloid leukemia
AUC: area under the curve
ALB: albumin ratio
CAL: coronary artery lesion
CAD: coronary artery dilatation
CAA: coronary artery aneurysm
CAS: coronary artery stenosis
CRP: C-reactive protein
DT: Decision Tree
Echo: Echocardiography
FPR: false positive rate
IVIG: intravenous immunoglobulin
IKS: incomplete KS
JCS: Japan Circulation Society
JPS: Japan Pediatric Society
KS: Kawasaki syndrome
KNN: K-nearest neighbor
MI: myocardial infarction
LR: logistic regression
NPV: negative predictive value
NT-proBNP: N-terminal-pro-brain natriuretic protein
PCA: principal component analysis
PPV: positive predictive value
PMN: polymorphonuclear Neutrophil
RF: random forest
ROC: receiver operating characteristic
STEMI: ST-segment elevation myocardial infarction
TPR: true positive rate
XGBoost: eXtreme Gradient Boosting
