# Supplementary material for: Prediction of coronary artery lesions in children with Kawasaki syndrome based on machine learning
Source: BMC Pediatr. 2024 Mar 5;24:158. doi: 10.1186/s12887-024-04608-2 (PMC10916227; doi:10.1186/s12887-024-04608-2)
Supplement: Supplementary file 1 — Supplementary Material 1: Numerical range of input features [file 12887_2024_4608_MOESM1_ESM.docx]

**Supplementary Table**

**Table S1 Numerical range of input features**

| **Input feature** | **Ranges** | **Input feature** | **Ranges** |
| --- | --- | --- | --- |
| Gender | 1/2 | WBC(×10^9^) | 9.39-20.51 |
| Height (cm) | 70.60-103.95 | Hb(g/L) | 97.59-125.45 |
| Weight (kg) | 5.78-19.98 | PLT(×10^9^) | 187.92-475.56 |
| Age (d) | 125.31-1434.51 | CRP(mg/L) | 17.47-117.73 |
| Hospitalization time (d) | 2.78-11.92 | ESR(mm/h) | 36.83-87.25 |
| Fever Time (d) | 3.12-9.06 | Na^+^(mmol/L) | 133.32-139.82 |
| Rash | 0/1/2 | ALT(U/L) | 15.58-423.78 |
| Bulbar conjunctival injection | 0/1 | AST(U/L) | 16.98-453.84 |
| Chapped lips | 0/1 | LDH(U/L) | 222.83-366.87 |
| Reddening of lips | 0/1 | CK(U/L) | 16.11-93.81 |
| Strawberry tongue | 0/1 | CK-MB(U/L) | 6.56-275.81 |
| Non-suppurative cervical lymphadenopathy | 0/1 | cTNI(ng/mL) | 0.13-4.53 |
| Changes of peripheral extremities | 0/1 | PCT(ng/ml) | 0.16-14.37 |
| Changes of Perineal | 0/1 | Diagnosis | 1/2/3 |
| Arrhythmia | 0/1 |  |  |

Gender: 1 male; 2 female. Rash: 0 none; 1 macular papule; 2 millet. Diagnosis: 1 KS; 2 IVIG-resistant KS; 3 IKS. For the rest of the features, 0 indicates that it has not occurred, and 1 indicates that it has occurred.
